# Pharmacological Properties of *Morus nigra* L. (Black Mulberry) as A Promising Nutraceutical Resource

**DOI:** 10.3390/nu11020437

**Published:** 2019-02-20

**Authors:** Sung Ho Lim, Chang-Ik Choi

**Affiliations:** College of Pharmacy, Dongguk University-Seoul, Goyang 10326, Korea; teruai0608@naver.com

**Keywords:** *Morus nigra* L., black mulberry, nutraceutical, pharmacological properties

## Abstract

Mulberry plants belonging to the Moraceae family have been grown for the purpose of being the nutrient source for silk worm and raw materials for the preparation of jams, marmalades, vinegars, juices, wines, and cosmetics. *Morus nigra* L. (black mulberry) is native to Southwestern Asia, and it has been used as a traditional herbal medicine for animals and humans. In this article, recent research progress on various biological and pharmacological properties of extracts, fractions, and isolated active constituents from different parts of *M. nigra* are reviewed. *M. nigra* exhibited a wide-spectrum of biological and pharmacological therapeutic effects including antinociceptive, anti-inflammatory, antimicrobial, anti-melanogenic, antidiabetic, anti-obesity, anti-hyperlipidemic, and anticancer activities. *M. nigra* also showed protective effects against various human organs and systems, mainly based on its antioxidant capacity. These findings strongly suggest that *M. nigra* can be used as a promising nutraceutical resource to control and prevent various chronic diseases.

## 1. Introduction

*Morus*, commonly known as mulberry, is the genus of a flowering plant belonging to the Moraceae family. They are widely distributed into subtropic regions of Asia (including Korea, Japan, China, and India), North America, and Africa [1]. In Asian countries, mulberry plants have been grown for the production of silk worms (*Bombyx mori* L.), because their leaves are a major and important nutrient source for silk worms [2]. Meanwhile, most European countries have usually used mulberry fruits to prepare jams, marmalades, vinegars, juices, wine, and cosmetic products [3]. Various parts of mulberry plants have also been used as traditional herbal medicines [4]. Diels-Alder-type adducts, flavonoids, benzofurans, stilbenes, and polyhydroxylated alkaloids are the most representative bioactive compounds identified from Sang-Bai-Pi (Chinese name for root barks of *Morus* species) [5]. Some previous review articles on *Morus alba* L. (*M. alba*), one of the most valuable plants rich in natural ingredients, have demonstrated that extracts, fractions and major constituents from *M. alba* exhibit numerous pharmacological activities such as antioxidant, anti-inflammatory, anticancer, antimicrobial, antifungal, skin-whitening, antidiabetic, anti-hyperlipidemic, anti-atherosclerotic, anti-obesity, cardioprotective, cognitive enhancing, hepatoprotective, anti-platelet, anxiolytic, anti-asthmatic, anthelmintic, antidepressant, and immunomodulatory activities [6,7,8].

*Morus nigra* L. (*M. nigra*), also called black mulberry, is native to Southwestern Asia. It has been grown throughout Europe and around the Mediterranean for centuries. Although biological and/or pharmacological activities of *M. nigra* have been relatively less studied compared to those of *M. alba*, several bioactive compounds isolated from *M. nigra* have also been used as herbal medicines for animals and humans due to their analgesic and anti-inflammatory effects [1]. Budiman et al. [9] briefly summarized chemical compounds isolated from various parts of *M. nigra* and their pharmacological activities. In this review article, we extensively covered recent research progress on biological and pharmacological properties of *M. nigra* extracts, fractions, and active constituents, suggesting its potential and usefulness as a nutraceutical resource. Major biological and pharmacological therapeutic activities of *M. nigra* were summarized in Table 1.

## 2. Antinociceptive Activity 

In 2000, de Souza et al. [10] firstly reported on the antinociceptive effect of morusin, the main prenylflavonoid of *M. nigra* isolated from acetonic extract of its root barks. Morusin showed a significant inhibitory effect on acetic acid-induced abdominal constriction responses and formalin-induced pain, and it also resulted in prolongation of the latency period in a hot plate test in mice. Because morusin is also purified from other mulberry plants, such as *M. alba* [11], *M. australis* [12] and *M. lhou* [13], this study result alone is insufficient to fully reflect the analgesic activity of *M. nigra*. Nine years later, Padilha et al. [14] investigated the antinociceptive effect of methylene chloride extract of *M. nigra* leaves in mice. Similar to the results of de Souza et al. [10], *M. nigra* leaves extract showed significantly and dose-dependently reduced acetic acid-induced writhing and formalin-induced pain and increased response latency period in a tail-immersion test and hot plate test without any acute toxicity when the dose of the extract was up to 300 mg/kg.

Two studies by Chen et al. [15,16] recently evaluated the antinociceptive properties of total flavonoid extracts and main active ingredients from fresh fruits of *M. nigra*. In the first study [15], total flavonoids from *M. nigra* showed dose-dependent decreases in the duration of formalin-induced pain-response behaviors. In the second study, three different mulberry fruits (*M. alba*, *M. nigra* and *M. mongolia*) were compared [16]. *M. nigra* fruits had more anthocyanin and flavonol contents than other species. The duration of the formalin-induced secondary pain phase (inflammatory phase) in the group treated with total flavonoid extract from *M. nigra* was significantly shorter than that in the control group. Reduced development of inflammatory cytokine interleukin-6 (IL-6) and an increased level of an anti-inflammatory cytokine IL-10 associated with the nuclear factor kappa-light-chain-enhancer of activated B cells (NF-κB) and nitric oxide (NO) pathways were observed after treatment with *M. nigra* extract, suggesting the possible mechanism of its antinociceptive effects. Interestingly, the three main flavonoid ingredients (cyanidin-3-*O*-glucoside, rutin and isoquercetin) from *M. nigra* did not reduce the duration of formalin-induced pain individually, although they significantly decreased such duration when they were used as a mixture.

## 3. Anti-Inflammatory Activity 

Inflammation is defined as a set of physiological defense mechanisms taking place in the body. However, inflammation is also considered an initial event of major chronic diseases such as cardiovascular, autoimmune, eye, age-related, neurodegenerative diseases, and cancers [17]. In this respect, inhibiting and controlling inflammatory responses in the human body can be one of fundamental approaches for treating chronic diseases.

As a follow-up research of a previous study on antinociceptive activity, Padilha et al. [18] evaluated the anti-inflammatory effects of methylene chloride extract of *M. nigra* leaves in male rats. *M. nigra* leaves extract significantly inhibited the volume of paw edema induced by intraplantar injection of carrageenan at a half-maximal inhibitory concentration (IC_50_) value of 15.2 mg/kg. *M. nigra* leaves also significantly inhibited the formation of granulomatous tissues in the chronic inflammation status using a cotton pellet-induced granuloma rat model (IC_50_ of 71.1 mg/kg). In the same year, Wang et al. [19] isolated three new compounds (mornigrol D, G and H) with six other known compounds (norartocarpetin, dihydrokaempferol, albanin A, albanin E, moracin M, and albafuran C) from the stem bark of *M. nigra* and assessed their anti-inflammatory activities by calculating the inhibition of releasing β-glucuronidase from rat polymorphonuclear leukocytes induced by platelet-activating factor. At a concentration of 10^−5^ M, mornigrol D and norartocarpetin showed potent anti-inflammatory properties, showing inhibition rates of 65.9% and 67.7%, respectively. In 2014, Zelová et al. [20] investigated into the anti-inflammatory activities of two Diels-Alder adducts (soroceal and sanggenon E) isolated from the root bark of *M. nigra*, by determining the attenuation of secretion of pro-inflammatory cytokines, tumor necrosis factor-alpha (TNF-α) and IL-1β, in lipopolysaccharide (LPS)-stimulated macrophages. Although sanggenon E significantly reduced the production of TNF-α compared to the vehicle control, both compounds failed to significantly affect the level of IL-1β.

Chen et al. [15] reported that the total flavonoid extract of *M. nigra* fruits can dose-dependently inhibit xylene-induced ear edema (edema rate 60.1% at a concentration of 200 mg/20 mL/kg) and carrageenan-induced paw edema (edema rate 9.5% at a concentration of 100 mg/20 mL/kg; 8.6% at a concentration of 200 mg/20 mL/kg) in mice. Levels of pro-inflammatory cytokines including IL-1β, TNF-α, NO, and interferon-gamma (IFN-γ) were also significantly decreased after the treatment of *M. nigra* fruit extract in mice with xylene-induced inflammation. In addition, *M. nigra* fruits extract significantly reduced levels of NO in LPS-stimulated RAW 264.7 cells without showing the cytotoxicity effect at the concentration of 50 to 100 μg/mL.

A very recent study [21] has shown that extracts of *M. nigra* pulps and leaves can improve survival rate and decrease the number of total leukocytes in bronchoalveloar lavage fluid in LPS-induced septic mice, indicating the reduction of inflammatory infiltrate in the lung. Although most hepatic and serum cytokine levels were not changed by the administration of *M. nigra* extracts, serum levels of TNF, an important mediator of sepsis, were significantly lower in the *M. nigra* extract-treated group than those in the septic animal group.

## 4. Antimicrobial Activity

Antibacterial activities of *M. nigra* leaves have been investigated in various organic fractions. Tahir et al. [22] reported that the ethyl acetate fraction of *M. nigra* leaves is active against four dental caries-causing bacterial strains: *Streptococcus mutans*, *Escherichia coli* (*E. coli*), *Staphylococcus aureus* (*S. aureus*), and *Bacillus subtilis* (*B. subtilis*). Also, the chloroform fraction showed antibacterial properties against *Pseudomonas aeruginosa* (*P. aeruginosa*) and *B. subtilis*, while the methanol fraction was only active against *B. subtilis*. No activity was observed for n-hexane or aqueous fraction. The inhibition rate of streptococcal biofilm formation (anti-adherence effect) by *M. nigra* ethyl acetate fraction was 87%. In another study conducted by Souza et al. [23], crude ethanol extract of *M. nigra* leaves exhibited bactericidal activities against *Bacillus cereus* (*B. cereus*), *Enterococcus faecalis* (*E. faecalis*), and *E. coli*, with minimal inhibitory concentration (MIC) and minimum bactericidal concentration (MBC) less than 0.195 mg/mL for all. Potent antibacterial activities against *B. cereus* and *E. faecalis* were also observed for hexane, chloroform and ethyl acetate extracts (MIC values < 0.195 mg/mL for all). However, their measured MBCs were over 6 mg/mL. It was noted that chloroform extract exclusively showed a bactericidal effect against *Salmonella choleraesuis* (MIC and MBC value < 0.195 mg/mL, respectively). The antibacterial activities of the total flavonoid extract of *M. nigra* fruits were evaluated against three inflammatory pain-causing bacteria, *E. coli*, *P. aeruginosa* and *S. aureus*. Its fruit extract strongly inhibited all three strains, with MBC values of 2 mg/mL or less [16].

The antimicrobial activities of fresh juice of *M. nigra* fruits against five Gram-positive and three Gram-negative bacterial strains have been compared with conventional antibiotics [24]. Although 100 μL of *M. nigra* fruits juice produced generally smaller zones of inhibition (ranging from 9.98 to 19.87 mm) than other antibiotics treated at their standard doses, it showed a broad-spectrum antimicrobial effect against both Gram-positive and Gram-negative bacteria, having the highest inhibition against *P. aeruginosa*. Minhas et al. [25] investigated into the antimicrobial effect of five *M. nigra* fruits extracts classified by different solvents against 16 bacterial and 2 fungal strains in comparison with conventional antibiotics and antifungal agent nystatin. Ethanolic and acetone extracts of *M. nigra* fruits showed highly-sensitive inhibition (defined as 20 mm or more longer diameter of zone of inhibition) against *E. coli*, *S. aureus*, and *Neisseria* spp.; methanolic extract against *Klebsiella pneumoniae* and *Neisseria* spp.; and chloroform extract against *Serratia marcesscens*, *Staphylococcus epidermidis* (*S. epidermidis*), *P. aeruginosa*, and *S. aureus*. Similar to the results of Khalid et al. [24], *M. nigra* extracts had smaller zones of inhibition than those observed with conventional drugs.

In a recent study assessing antibacterial activities against two strains causing acne, *S. epidermidis* and *Propionibacterium acnes* (*P. acnes*), the ethanolic extract of *M. nigra* fruits had MIC values of 2.5% for both strains and MBC values of 2.5% and 5%, respectively [26]. As a follow-up approach, a comparative study was performed for extracts from three parts (stem barks, fruits and leaves) of *M. nigra* on their antibacterial effects against *S. epidermidis* and *P. acnes* [27]. *M. nigra* stem barks possessed the most potent antibacterial activities against both strains, with an MIC value of 4 mg/mL for *S. epidermidis* and 2 mg/mL for *P. acnes*. In addition, *M. nigra* stem barks extract induced nucleic acid, protein, and ion leakages and cellular membrane damages against *P. acnes*. These results suggest that the antibacterial effect of *M. nigra* stem bark is related to reduced cell membrane fluidity and bacterial cell wall destruction.

Mazzimba et al. [28] reported that six isolated constituents (oxyresveratrol, moracin M, cyclomorusin, morusin, kuwanon C, and a derivative of kuwanon C) from aerial parts of *M. nigra* show antibacterial activities against *S. aureus*, *B. subtilis*, *Micrococcus flavus*, *S. faecalis*, *Salmonella abony*, and *P. aeruginosa*, with morusin having the most potent activity against *B. subtilis* (MIC value 3.91 μg/mL).

Tuberculosis (TB), an infectious disease caused by *Mycobacterium tuberculosis* (*M. tuberculosis*), is one of the top 10 causes of death in the world. TB is a curable and preventive disease, but resistance against conventional antibiotic medications for *M. tuberculosis* has increased the number of cases of multidrug-resistant or extensively drug-resistant TB [29]. In this respect, demand for new medications with novel therapeutic targets such as protein tyrosinase phosphatases (PTPs) is growing [30,31]. Mascarello et al. [32] evaluated the anti-tuberculosis activity of Diel–Alder-type adducts from *M. nigra* root bark to determine their potential as candidates for *M. tuberculosis* PTP inhibitor. A total of eight compounds (Kuwanon L, G, and H; cudraflavanone A; morusin, oxyresveratrol; chalcomoracin; and norartocarpetin) were isolated from *M. nigra*. They all significantly inhibited *M. tuberculosis* PTP-B (Mtb PtpB) with IC_50_ values ranging from 0.36 to 8.42 μM. Further enzyme kinetic analyses for Kuwanon G and H, two of the most potent compounds, showed that both compounds competitively inhibited Mtb PtpB, with inhibitory constant (K_i_) values of 0.39 ± 0.27 μM and 0.20 ± 0.01 μM, respectively. In addition, Kuwanon G inhibited the growth of *M. tuberculosis* inside macrophages by 61.3% at a non-cytotoxic concentration (10 μg/mL, corresponding to 14.4 μM of Kuwanon G), indicating that it is the most promising anti-tuberculosis constituent isolated from *M. nigra*.

Antimicrobial activity of *M. nigra* against *Candida* spp., the most common cause of fungal infections around the world [33], was assessed with aqueous and methanol extracts of its fruits, by using a disc-diffusion assay [34]. Of nine selected *Candida* spp., both extracts exhibited anticandidal effect against *Candida* (*C.*) *albicans*, *C. parapsilosis*, *C. tropicalis*, and *Geotricum candidum*, with lower MIC values observed for the methanol extract (0.625–2.5 mg/mL) than those for the aqueous extract (1.25–5 mg/mL).

## 5. Anti-Melanogenic (Skin-Whitening) Activity

Although melanin pigmentation in the skin is an important defense mechanism against ultraviolet radiation, abnormal melanin hyperpigmentation catalyzed by tyrosinase can cause several serious aesthetic problems [35,36,37]. As an anti-melanogenic strategy, tyrosinase inhibitors have become increasingly important for treating skin disorders associated with pigmentation and to improve skin-whitening.

Zhang et al. [38] investigated the inhibitory effect of 2,4,2’,4’-tetrahydroxy-3-(3-methyl-2-butenyl)-chalcone (TMBC) isolated from the stem of *M. nigra* on tyrosinase activity and melanin biosynthesis. TMBC dose-dependently and competitively inhibited mushroom tyrosinase-mediated L-dopa oxidation (IC_50_ value 0.95 ± 0.04 μM), which was more potent than kojic acid (IC_50_ value 24.88 ± 1.13 μM), a well-known skin depigmenting agent. Furthermore, TMBC significantly reduced the melanin content and cellular tyrosinase activity in B16 melanoma cells, although it increased mRNA levels of cellular tyrosinase. Zheng et al. [39] screened tyrosinase inhibitory properties of a total of 29 constituents isolated from roots of *M. nigra*. Among them, nine compounds (5’-geranyl-5,7,2’,4’-tetrahydroxyflavone, steppogenin-7-*O*-β-d-glucoside, 2,4,2’,4’-tetrahydroxychalcone, moracin N, kuwanon H, mulberrofuran G, morachalcone A, oxyresveratrol-3’-*O*-β-d-glucopyranoside and oxyresveratrol-2-*O*-β-d-glucopyranoside) showed better tyrosinase inhibitory activities than kojic acid (IC_50_ value 46.95 ± 1.72 μM, with 2,4,2’,4’-tetrahydroxychalcone having the highest activity (IC_50_ value 0.062 ± 0.002 μM, 757-fold lower IC_50_ than kojic acid). More recently, de Freitas et al. [40] reported that five different batches of standardized ethanolic extracts of *M. nigra* leaves all exhibited tyrosinase inhibitory activities, with IC_50_ ranging from 5.00 to 8.49 μg/mL.

Koyu et al. [41] tested the microwave-assisted extraction of fresh fruits of *M. nigra* in variable conditions for optimizing and maximizing tyrosinase inhibitory activity. Consequently, the highest tyrosinase inhibitory activity (IC_50_ value 1.44 mg/mL) was observed in the optimum microwave extraction system yielding the highest amount of anthocyanin content (13.28 mg/g cyanidin-3-glucoside equivalent), suggesting the important potential of anthocyanins on tyrosinase inhibition.

## 6. Antidiabetic and Anti-Obesity Activity

Diabetes mellitus is a chronic endocrine disorder characterized by hyperglycemia related to metabolic impairment of insulin production, secretion, and/or utilization. It is closely associated with the development of several important complications in cardiovascular, neurological and renal systems that can lead to increased morbidity and mortality in diabetic patients [42]. Various classes of antihyperglycemic agents are now available. However, some undesirable adverse effects such as hypoglycemia, gastrointestinal symptoms, weight gain and hepato-renal toxicity caused by the administration of these medications have been arousing interests on the discovery of new effective and safer naturally-occurring antidiabetic agents with different therapeutic pathophysiological mechanisms and targets [43,44,45].

*M. nigra* has also shown good antidiabetic effects on extracts and active constituents from some parts of this plant. Abd El-Mawla et al. [46] investigated the hypoglycemic efficacy of *M. nigra* leaf extracts and its cell suspension cultures treated with methyl jasmonate to induce accumulation of flavonoid contents in cell cultures. Extracts from *M. nigra* leaves dose-dependently decreased plasma glucose concentrations and increased insulin levels up to 500 mg/kg/day in streptozotocin (STZ)-treated diabetic rats. In addition, a slightly higher hypoglycemic effect was observed when rats were treated with extracts from cultured cells, indicating the additive action of flavonoids induced by methyl jasmonate. Hydroethanolic extracts of *M. nigra* leaves also significantly decreased serum fasting and 2-h glucose concentrations (at dose of 50 mg/kg) and increased serum insulin level (at dose of 10 mg/kg) in nicotinamide-STZ-induced type 2 diabetic rats [47]. Diabetes-induced changes in blood vessels may enhance the pathophysiological activity of metalloproteinases (MMPs). It is known that the inhibition of MMPs can improve insulin resistance and oxidative stress [48,49]. Araujo et al. [49] demonstrated the hypoglycemic potential of *M. nigra* leaves via reduction of expression and activity of MMP-2 in livers of diabetic rats. In addition, several phenolic compounds and isoprenylated flavonoids isolated from extracts of *M. nigra* twigs showed good antidiabetic activities, involving mechanisms of peroxisome proliferators-activated receptor gamma (PPARγ) activation [50] and α-glucosidase inhibition [51]. On the other hand, 3-week treatment of aqueous extract of *M. nigra* leaves failed to affect serum glucose levels in non-diabetic or diabetic pregnant rats [52].

Although there is no published report on the antidiabetic activity of black mulberry fruit yet, its effects on obesity, associated with increased risk of many chronic adverse health effects including cardiovascular diseases, dyslipidemia, non-alcoholic hepatic disease, cancer, and type 2 diabetes [53,54] have been evaluated by Fabroni et al. [55]. They demonstrated that 80% hydroethanolic freeze-dried extract of fruits of *M. nigra* had moderate total anthocyanin and total phenolic contents, with an IC_50_ value for pancreatic lipase inhibition at 6.32 ± 0.01 mg/mL. 

## 7. Anti-Hyperlipidemic and Anti-Atherosclerotic Activity

Cholesterol is a lipid molecule that acts as a structural component of cell membrane modulating fluidity and permeability, and as a precursor for steroid hormone and bile acid synthesis [56]. At the same time, hypercholesterolemia, a typical type of hyperlipidemia characterized by excessive accumulation of cholesterol in serum, is one of the crucial risk factors for coronary heart disease and atherosclerotic progression [57]. It has also been reported that reduction of low-density lipoprotein cholesterol (LDL-C) and improvement in levels of high-density lipoprotein cholesterol (HDL-C) can contribute to the anti-atherogenic condition [58,59].

Results from biochemical profile studies conducted by Volpato et al. [52] and Mahmoud [60] demonstrated that *M. nigra* extracts can decrease total cholesterol, triglyceride, LDL-C, and very low-density lipoprotein cholesterol (VLDL-C) levels and increase HDL-C in diabetic pregnant rats [52] and rats fed a high-fat diet [60]. Zeni et al. [61] evaluated the lipid-lowering effect of *M. nigra* leaf extract using Triton WR-1339-induced hyperlipidemic rats. The LDL-C level had significantly decreased after treatment with 100 mg/kg *M. nigra* infusion extract and HDL-C levels were restored in all groups treated with *M. nigra* extract at three different concentrations (100, 200 and 400 mg/kg), compared to those in the group only treated with Triton WR-1339. Atherogenic index and cardiac risk factor, indicators of likelihood of cardiovascular diseases associated with hyperlipidemia, were also decreased by *M. nigra* leaf extract. In another study by Jiang et al. [62], a high dose (210 mg/kg) of ethanolic extract of *M. nigra* fruit (EEBM) resulted in lowering mean body weight in rats fed a 6-week high-fat diet, which is comparable to the effect observed in the group treated with 5 mg/kg simvastatin. EEBM also dose-dependently improved serum lipid profiles, atherosclerosis indexes and lipid peroxidation compared to the control (high-fat diet-induced hyperlipidemic model) group. Histopathological changes in rat liver and thoracic aorta with reduction in the intima-media thickness of rat aortic arch after treatment with EEBM suggest that *M. nigra* fruit can effectively suppress the development and deterioration of atherosclerosis.

## 8. Organ-Protective Activity

### 8.1. Neuroprotective Effect

Turgut et al. [63] investigated the effect of *M. nigra* leaves extract on D-galactose-induced cognitive impairment and oxidative stress in mice. The results from the Morris water maze test showed significant and dose-dependent decreases in mean escape latency and time required to reach the target quadrant. Time spent in the target quadrant and number of times crossed the platform location were increased after the administration of lyophilized *M. nigra* extract, suggesting its potential neuroprotective role by preventing D-galactose-induced learning dysfunction and memory loss. *M. nigra* extract also showed DNA damage protection, reduced malondialdehyde (MDA) levels and augmented activities of three anti-oxidant enzymes, superoxide dismutase (SOD), glutathione peroxidase (GPx), and catalase (CAT) in the serum, brain and liver of D-galactose-treated mice. These antioxidant and anti-aging properties are considered as one of key mechanisms of *M. nigra* in delaying neurodegenerative processes.

Dalmagro et al. [64] performed a forced swimming test (FST) and tail suspension test (TST) to evaluate antidepressant-like activities of *M. nigra* and its major phenolic compounds syringic acid in mice. Acute and subchronic oral administration of aqueous extract of *M. nigra* leaves significantly decreased the immobility time in FST and TST except for acute administration at a dose of 100 mg/kg extract in TST. Acute treatment with 1 mg/kg and 10 mg/kg and subchronic treatment with 1 mg/kg of syringic acid also significantly decreased immobility time in TST. Nitro-oxidative stress in the serum and brain was assessed by measuring thiobarbituric acid reactive substances (TBARS), nitrite, protein carbonyl content (PC) and non-protein thiol groups (NPSH) levels, with some inconsistent and controversial study results. A significant decrease of TBARS level was observed at acute doses of 3 mg/kg *M. nigra* extract. However, TBARS levels were oppositely increased at subchronic doses of 3, 10, and 100 mg/kg extract in the serum and at a subchronic dose of 3 mg/kg extract in the brain. Levels of nitrites in the serum were significantly decreased after subchronic administration of 10, 30 and 100 mg/kg extracts of *M. nigra* leaves, and nitrites in the brain were also decreased after subchronic treatment with the extract at doses of 30 and 100 mg/kg. In addition, subchronic treatment with 1 mg/kg syringic acid resulted in significant changes in TBARS and nitrite levels in the serum and brain (all decreased, except TBARS level was increased in the brain). PC level was decreased after treatment with 30 mg/kg *M. nigra* extract and syringic acid. There was no significant change in NPSH level at all treatment conditions. Nevertheless, *M. nigra* leaf extract and syringic acid both exhibited good cell viabilities in hippocampal and cerebral cortex slices incubated with 100 mM glutamate, suggesting their proper neuroprotective effect against glutamate-induced toxicity. 

### 8.2. Hepatoprotective Effect

Tag et al. [65] evaluated the hepatoprotective effect of the ethanolic extract of *M. nigra* leaves. With an IC_50_ value at 14.5 μg/mL in in vitro cytotoxicity to HepG2 (a well-differentiated human hepatocellular carcinoma) cell line, *M. nigra* leaf extract also significantly decreased levels of liver enzymes alanine aminotransaminase (ALT), aspartate aminotransaminase (AST), alkaline phosphatase (ALP), and lactate dehydrogenase (LDH) in male albino rats with methotrexate-induced hepatotoxicity. Hematosomatic index, defined as the ratio between liver- and body-weight and considered as an indicator for hepatic damage and liver inflammation, in the group co-treated with *M. nigra* extract and methotrexate, was also apparently decreased compared to that in methotrexate-only treated group. In histopathological studies, *M. nigra* treatment resulted in moderate enhancement in the hepatoprotection from methotrexate-related injury. Microscopic damage scores (hepatocyte degeneration, congestion, leukocyte infiltration, fibrosis, and total histopathology score) were significantly decreased when *M. nigra* extract was simultaneously administered compared to those in the group treated with methotrexate alone. In addition, methotrexate-induced progressive increases in collagen deposition of liver tissue were normalized by treatment with *M. nigra* leaf extract. Another study performed by Hassanalilou et al. [66] also showed that *M. nigra* leaf extract can lead to less fatty degeneration in liver tissue and smaller distension of hepatic cytoplasm due to fatty droplets in STZ-induced diabetic rats along with reduced fasting blood glucose, compared to glibenclamide, a well-known sulfonylurea antihyperglycemic agent.

Hepatoprotective activity of *M. nigra* fruits in carbon tetrachloride (CCl_4_, a well-known potent hepatotoxin)-treated HepG2 cells [67] and adult male Sprague-Dawley rats [68] have been reported. Extracts of *M. nigra* fruits dose-dependently and significantly reduced levels of hepatic enzymes AST, ALT and gamma-glutamyl transferase (GGT) compared to control (CCl_4_-treated group). At the same time, they significantly increased SOD and gluatathione peroxidase (GPx) enzymatic capacities and decreased expression levels and activities hepatic capsase-3 (a biomarker for cell apoptosis) and 8-oxo-2’-deoxyguanosine (a biomarker for oxidative stress) in rat liver tissues, indicating that the hepatoprotective effect of *M. nigra* fruits might be closely associated with its antioxidant activity [67,68].

### 8.3. Renal-Protective Effect

The effects of hydroalcoholic extract of *M. nigra* fruits on biochemical and histopathological changes in serum and kidney tissues have been evaluated in alloxan-induced diabetic rats [69]. Milder glomerular damage and no mesenchymal tissue expansion into renal glomerular vessels were observed in the group after 8 weeks of treatment with 800 mg/kg *M. nigra* fruit extract compared to those in diabetic and positive control (150 mg/kg metformin) groups. Although an increase in serum creatinine level was observed in the group treated with 800 mg/kg *M. nigra* extract, this group had lower serum glucose and urea levels compared to diabetic and positive control groups. These results suggest that *M. nigra* fruits have a protective effect on diabetic nephropathy and related kidney tissue injury. The extract of *M. nigra* leaves also significantly improved biochemical parameters reflecting kidney functions (serum creatinine, urea, and uric acid) and exhibited milder histopathological glycogen accumulation, fatty degeneration, and lymphocyte infiltration of renal convoluted tubules in STZ-induced diabetic rats compared to non-treated and glibenclamide-treated groups [66].

### 8.4. Gastroprotective Effect

Nesello et al. [70] reported that oral administration of methanolic extract from *M. nigra* fruits at a high dose (300 mg/kg) can protect gastric mucosa against acidified ethanol-induced acute gastric ulcer in female mice. This study result was confirmed by macroscopic and microscopic representative images, showing that the degree of epithelial damage in gastric tissue was decreased. To further investigate the underlying mechanisms for the gastroprotective effect, levels of lipid hydroperoxide (LOOH) and glutathione (GSH) in ulcerated gastric mucosa were quantified. *M. nigra* fruits extract prevented GSH depletion and promoted partial reduction of LOOH, suggesting its ability to ameliorate oxidative stress involved in the development of gastric injury by acidified ethanol. Because *M. nigra* fruits did not affect the activity of H^+^/K^+^-ATPase in their study, they have pharmacological advantages of being free from the risk of several side effects such as rebound acid hypersecretion, hypergastrinemia, gastric polyps, or atrophic gastritis [71] known to be associated with suppressed gastric acid secretion.

## 9. Activity on Female Reproductive System

De Queiroz et al. [72] investigated the estrogenic effect of *M. nigra* on the female reproductive system and embryonic development. Five different concentrations (25, 50, 75, 350, and 700 mg/kg) of hydroalcoholic extract of dried *M. nigra* leaves were administered in female Wistar rats for 15 days and their biological and clinical features were compared with the control group, in which distilled water instead of *M. nigra* extract was used as treatment. There were no significant differences in the number of deaths, clinical signs of toxicity, changes in food consumption, or body weight between groups, suggesting that *M. nigra* leaves did not cause maternal reproductive toxicity. Histological changes in ovarian structures, signs of edema, cystic follicles, retained oocytes, or thickened uterine epithelium were not observed. The number of corpora lutea, live fetuses, implants, resorptions, implantation, and pre- or post-implantation loss were not affected by the administration of *M. nigra* leaf extract either. Consequently, *M. nigra* exhibited no estrogenic effect or toxicity on the female reproductive system.

Another study conducted by Cavalcante et al. [73] showed that ethanolic extract of *M. nigra* fresh leaves at 0.1 mg/mL can improve percentages of follicular morphology, antrum formation, and fully grown oocytes, as well as the diameter of follicles compared to control group at 12 days after treatment. Furthermore, additive effects on follicular growth (described as follicular diameter increase and higher daily growth rate) were observed when *M. nigra* extract with supplemented medium and follicle-stimulating hormone (FSH) were used as co-treatment, indicating its capacity on ovine secondary follicle development.

## 10. Anticancer Activity

Cancer is a life-threatening disease state characterized by unregulated and permanent cell growth and proliferation [74]. Because of its ability to avoid programmed cell death (apoptosis) as one of the main driving forces for maintaining cancer cell proliferation, induction of apoptosis in cancer has been considered a reasonable strategy to treat cancer [75,76].

Morniga M, a mannose-specific jacalin-related lectin from the bark of *M. nigra*, can preferentially trigger the proliferation and activation of human T- and natural killer- (NK-)lymphocytes and dose-dependently induce cell death of α-CD3 activated T lymphocytes when compared with concanavalin A (Con A), a well-known mannose-specific legume leptin from *Canavalia ensiformis* [77]. Results from flow cytometry analysis have demonstrated that morniga M-induced cell death is probably associated with the apoptotic mechanism, suggesting the anticancer potential of morniga M via cell-death induction and immunomodulation as reported in previous studies with Con A [78,79]. Anticancer activities of morniga M were further investigated by Çakıroğlu et al. [80], in which they demonstrated that both *M. nigra* fruit extract and morniga M significantly and dose-dependently decreased cell viability against HT-29 cell line (human colorectal cancer). Another brief research by Qadir et al. [81] the demonstrated dose-dependent anticancer activity of n-hexane and aqueous methanol extract of *M. nigra* leaves against HeLa cell line (human cervical cancer), with IC_50_ values of 185.9 ± 8.3 µg/mL and 56.0 ± 1.7 µg/mL, respectively.

Anti-proliferative and apoptotic effects of *M. nigra* fruits against several human adenocarcinoma cell lines have been reported [80,82,83]. Ahmed et al. [82] compared the anticancer effects between fresh and dried fruit extracts of *M. nigra* on MCF-7 cell line (human breast cancer). Study results have shown that both ethanolic extracts dose- and time-dependently inhibit cellular growth of MCF-7 cells; exhibit apoptotic morphological changes in their cytoplasmic membranes, cell bodies, and nuclei; induce DNA fragmentations and single strand breaks; and decrease mitotic indexes, with better pharmacological properties in fresh fruit of *M. nigra*. Turan et al. [83] evaluated the anticancer activities of *M. nigra* fruit extract on PC-3 cells (human prostate cancer). Dimethyl sulfoxide (DMSO) extract of *M. nigra* exhibited moderate cytotoxicity against PC-3 cells with an IC_50_ value of 370.1 ± 5.8 μg/mL. It significantly increased the cell number at G_0_/G_1_ phase and decreased the cell number at S phase, indicating that *M. nigra* fruits inhibited the progression of the cell cycle at the G_0_/G_1_ phase. *M. nigra* fruit extract at a high dose (666 μg/mL) significantly increased the number of necrotic, early apoptotic and late apoptotic cells compared to the untreated control group. It also dose-dependently decreased mitochondrial membrane potential and increased activities of caspase 3 and 7 (key mediators of apoptosis) in PC-3 cells [83].

## 11. Antioxidant Activity

Oxidative stress is characterized by an excessive increase in intracellular oxidizing species such as reactive oxygen species (ROS) involved in the loss of antioxidant defense capacity. It plays a critical role in various clinical conditions including aging, cancer, diabetes, atherosclerosis, chronic inflammation, neurodegenerative diseases, rheumatoid arthritis, human immunodeficiency virus (HIV) infection, ischemia and reperfusion injury, and obstructive sleep apnea [84,85]. Many researchers are interested in the antioxidant activity of naturally-occurring ingredients because phenolic compounds and flavonoids, the largest phytochemical molecules from natural resources, possess a variety of biological properties including antioxidant activity [86,87,88,89]. It has also been widely reported that mulberries are rich in anthocyanin constituents having remarkable antioxidant activities and other health benefits such as anti-inflammatory, antimicrobial, anti-obesity, antidiabetic, anti-hyperlipidemic, antihypertensive, cardioprotective (reduced risk of coronary heart disease and stroke), and anticancer effects [90,91,92].

Numerous researches have proven antioxidant properties of *M. nigra* with different in vitro methods, including DPPH (2,2-diphenyl-1-picrylhydrazyl) radical scavenging assay [15,23,24,25,26,28,61,70,93,94,95,96,97,98,99,100,101,102,103,104,105,106,107,108,109,110,111,112,113], ABTS (2,2’-azino-bis(3-ethylbenzothiazoline)-6-sulfonic acid) radical scavenging assay [15,91,94,99,100,101,102,103,107,110,112,113,114,115,116,117], reducing power assay [15,99,113,118,119], superoxide anion radical (O2^−^) scavenging assay [15,118,120], hydroxyl radical (OH-) scavenging assay [15,113,120], lipid peroxidation assay [19,52,60,62,63,64,70,121,122], antioxidant enzyme activity assay [21,49,52,62,63,67], β-carotene bleaching assay [23,119,123], ferric-reducing antioxidant power (FRAP) assay [24,85,91,95,100,102,104,107,110,111], protein carbonyl assay [49,64,96], GSH measurement [67,70,112], hydrogen peroxide (H2O2)-induced injury assay [113,121], NO radical scavenging assay [111,118], SOD-like activity [96], cupric-ion reducing antioxidant capacity (CUPRAC) assay [102,107,108,110], H_2_O_2_ scavenging assay [108,119], phosphomolybdenum assay [108,119], and ROS measurement [112].

## 12. Other Pharmacological Activities

Malik et al. [124] investigated the cardiovascular activity of aqueous methanolic extract of *M. nigra* fruit in frogs. Treatment of *M. nigra* fruit extract showed significant and dose-dependent decreases in heart rate without direct effects on the contractility of frog’s heart. Results of phytochemical analysis revealed the presence of cardiac glycosides in *M. nigra* fruit, along with other active constituents including saponins, alkaloids, phenolic compounds, and flavonoids.

Crude extract and fractions of *M. nigra* fruits exhibit both in vitro and in vivo prokinetic, laxative, and antidiarrheal effects [125]. *M. nigra* extract significantly promoted the transit of charcoal meal through the small intestine, increased gastric emptying rate and the mean number of wet feces, and decreased castor oil-induced diarrhea in mice. In in vitro studies, chloroform and petroleum ether fractions of *M. nigra* fruits dose-dependently inhibited carbachol- and potassium ion-induced contractions of rabbit jejunum while aqueous and ethyl acetate fractions showed stimulatory effects on guinea-pig ileum. Suppression of maximum responses of acetylcholine and calcium ion (Ca^2+^) by *M. nigra* fruits was also observed, and most gastrointestinal effects were conversely affected by concomitant administration of atropine, suggesting that the underlying mechanisms of these prokinetic, laxative, and antidiarrheal activities might be associated with cholinergic control and Ca^2+^ channel antagonism [125].

Fahimi and Jahromy [126] described the effects of *M. nigra* fruit juice on levodopa-induced dyskinesia in mice with 1-methyl-4-phenyl-1,2,3,6-tetrahydropyridine (MPTP)-induced Parkinson’s disease. After 14 days of levodopa treatment, administration of 10 or 15 mL/kg of *M. nigra* fruit juice significantly decreased abnormal involuntary movement scale (AIMS) scores compared to levodopa treatment only.

## 13. Drug-Food Interaction and Toxicity

Food ingredients can cause drug-food interactions, most of which are pharmacokinetic interactions associated with the alteration in activities of drug-metabolizing enzymes or drug transporters [127]. A brief experimental report by Kim et al. [128] demonstrated that the fruit juice of *M. nigra* has a potent inhibitory effect of human liver microsomal cytochrome P450 3A (CYP3A) activity, with IC_50_ values for midazolam (a probe drug for CYP3A) 1‘-hydroxylation of 2.96 ± 0.33% (*v*/*v*, with 20-min preincubation) and 6.22 ± 0.47% (no preincubation). Because approximately 30% of clinically used drugs including macrolide antibiotics, antiarrhythmics, benzodiazepines, immune modulators, human immunodeficiency virus (HIV) antivirals, antihistamines, calcium channel blockers, and statins are metabolized by CYP3A [129,130], concomitant intake of CYP3A substrates with *M. nigra* fruit can lead to an increase in plasma drug exposure.

Figueredo et al. [131] assessed the acute and subacute toxicities of *M. nigra* leaves extract in Wistar rats. A single or 28-day oral dose of ethanolic extract of *M. nigra* leaves did not cause any adverse effects. It did not induce abnormal behaviors or mortality. *M. nigra* extract resulted in some significant but non-toxic changes in biochemical profiles (decreased urea and AST in males; decreased total cholesterol and AST in females) and leukocyte parameters (increased neutrophils in males; decreased white blood cell in females). *M. nigra* leaves did not affect lipid peroxidation and changes in renal and hepatic CAT enzymatic activities.

**Table 1:** Summary of major biological and pharmacological therapeutic activities of *M. nigra*.

Pharmacological Activity	Study Model	Used Part	SampleType ^a^	Ref.
Antinociceptive	Swiss mice	Root bark	C	[10]
	Male Swiss mice	Leaf	E	[14]
	Male Kunming mice	Fruit	E	[15]
	Male Kunming mice	Fruit	E,C	[16]
Anti-inflammatory	Kunming male mice; RAW 264.7 cell	Fruit	E	[15]
	Adult male rats	Leaf	E	[18]
	Rat polymorphonuclear leukocytes	Bark	C	[19]
	THP-1 human monocytic leukemia cell line	Root	C	[20]
	Male C57BL/6 mice	Pulp; leaf	E	[21]
Antimicrobial	In vitro assay	Fruit	E	[16]
	In vitro assay	Leaf	E,F	[22]
	In vitro assay	Leaf	E,F	[23]
	In vitro assay	Fruit	J	[24]
	In vitro assay	Fruit	E	[25]
	In vitro assay	Fruit	E	[26]
	In vitro assay	Stem bark; fruit; leaf	E	[27]
	In vitro assay	Stem bark; stem wood	E,C	[28]
	In vitro assay; THP-1 cell line	Root	E,C	[32]
	In vitro assay	Fruit	E	[34]
Anti-melanogenic (Skin-whitening)	In vitro assay; B16 melanoma cells	Stem	C	[38]
	In vitro assay	Root; twig	C	[39]
	In vitro assay	Leaf	E	[40]
	In vitro assay	Fruit	E	[41]
Antidiabetic	Male Wistar rats	Leaf	E ^b^	[46]
	Male albino mice	Leaf	E	[47]
	Female albino Fischer rats	Pulp; leaf	E	[49]
	PPARγ-transfected HEK293 cells	Twig	C	[50]
	In vitro assay	Twig	C	[51]
Anti-obesity	In vitro assay	Fruit	E	[55]
Anti-hyperlipidemic	Wistar rats	Leaf	E	[52]
	Adult male albino Sprague-Dawley rats	Fruit	E	[60]
	Male Wistar rats	Leaf	E	[61]
	Male Spraque-Dawley rats	Fruit	E	[62]
Organ-protective	Male BALB/c mice	Leaf	E	[63]
	Male Swiss mice	Leaf	E,C	[64]
	HepG2 human hepatocellular carcinoma cell line; male albino rats	Leaf	E	[65]
	Male Wistar rats	Leaf	E	[66]
	HepG2 cells	Fruit	E	[67]
	Adult male Sprague-Dawley rats	Fruit	E	[68]
	Male Wistar rats	Fruit	E	[69]
	Female Swiss mice	Fruit	E	[70]
Anticancer	Peripheral blood mononuclear cells (PBMCs); peripheral blood T lymphocytes; Jurkat T leukemia cells	Bark	C	[77]
	HT-29 human colorectal adenocarcinoma cell line	Fruit	E,C	[80]
	HeLa human cervical cancer cell line	Leaf	E	[81]
	MCF-7 human breast cancer cell line	Fruit	E	[82]
	PC-3 human prostate adenocarcinoma cells	Fruit	E	[83]

^a^, E, extract; F, fraction; C, isolated compound; J, juice. ^b^, Cell suspension cultures of *M. nigra* extract were used.

## 14. Conclusions

*M. nigra*, especially its leaf and fruit parts, exhibited various pharmacological properties including antinociceptive, anti-inflammatory, antimicrobial, anti-melanogenic, antidiabetic, anti-obesity, anti-hyperlipidemic, and anticancer activities. *M. nigra* also showed protective and therapeutic effects on the central nervous system, liver, kidney, gastrointestinal tract, and female reproductive system. Most of these features were attributable to its antioxidant capacity due to abundant phytochemical constituents such as polyphenols, flavonoids and anthocyanins. These findings suggest that *M. nigra* can be used as a promising nutraceutical resource to control and prevent various chronic diseases. Given that most researches are performed in vitro and in animal models, further studies at the clinical level are required to establish the efficacy and safety of *M. nigra* in the human body.
